# The Strange Case of Functional High-Risk Multiple Myeloma Patients: Is It Possible to Identify Them in Clinical Practice?

**DOI:** 10.3390/cancers17213580

**Published:** 2025-11-06

**Authors:** Sonia Morè, Massimo Offidani, Laura Corvatta, Tommaso Za, Francesca Fazio, Martina Gherardini, Velia Bongarzoni, Barbara Anaclerico, Luca Franceschini, Silvia Ferraro, Luca Cupelli, Carmine Liberatore, Laura De Padua, Angela Rago, Silvia Gentili, Roberto Latagliata, Mariagrazia Garzia, Iole Cordone, Valeria Mezzanotte, Elena Rossi, Francesca Di Landro, Maria Zaira Limongi, Erika Morsia, Antonella Poloni, Maria Teresa Petrucci

**Affiliations:** 1 Department of Clinical and Molecular Sciences, Dipartimento Scienze Cliniche e Molecolari (DISCLIMO), Università Politecnica delle Marche, 60121 Ancona, Italy; s.more@staff.univpm.it (S.M.); e.morsia@staff.univpm.it (E.M.); a.poloni@univpm.it (A.P.); 2 Hematology Clinic, AOU delle Marche, 60126 Ancona, Italy; 3 Internal Medicine, Ospedale E. Profili, 60044 Fabriano, Italy; laura.corvatta@sanita.marche.it; 4 Hematology Clinic, Policlinico Agostino Gemelli, 00136 Rome, Italy; toza251978@yahoo.it (T.Z.); elena.rossi@unicatt.it (E.R.); francesca.dilandro@guest.policlinicogemelli.it (F.D.L.); 5 Hematology Department of Translational and Precision Medicine, Azienda Policlinico Umberto I, Sapienza University of Rome, 00161 Roma, Italy; fazio@bce.uniroma1.it (F.F.); martina.gherardini@uniroma1.it (M.G.); mariazairalimongi@yahoo.it (M.Z.L.); petrucci@bce.uniroma1.it (M.T.P.); 6 Department of Hematology, San Giovanni-Addolorata Hospital, 00184 Rome, Italy; vbongarzoni@hsangiovanni.roma.it (V.B.); banaclerico@hsangiovanni.roma.it (B.A.); 7 Lymphoproliferative Diseases Unit, Tor Vergata University Hospital, 00133 Rome, Italy; luca.franceschini@ptvonline.it (L.F.); valeria.mezzanotte@ptvonline.it (V.M.); 8 Hematology Department, Ospedale Sant’Eugenio, 00144 Rome, Italy; silvia.ferraro@aslroma2.it (S.F.); luca.cupelli@gmail.com (L.C.); 9 Hematology Unit, Pescara Hospital, 65124 Pescara, Italy; carmine.liberatore@asl.pe.it; 10 UOC di Ematologia, Trapianto di Cellule Staminali e Terapia Genica, 03100 Frosinone, Italy; laura_dp_81@libero.it; 11 UOSD Ematologia ASL Roma1, 00193 Rome, Italy; angela.rago@aslroma1.it; 12 UOSD Ematologia Civitanova Marche AST Macerata, 62012 Civitanova Marche, Italy; silvia.gentili@sanita.marche.it; 13 UOC Hematology, Santa Rosa Hospital, 01100 Viterbo, Italy; roberto.latagliata@asl.vt.it; 14 Department of Hematology, Hematology San Camillo Forlanini Hospital, 00152 Rome, Italy; mgarzia@scamilloforlanini.rm.it; 15 IRCCS Istituto Nazionale Tumori Regina Elena Dipartimento di Ricerca, Diagnostica Avanzata ed Innovazione Tecnologica-IFO, 00144 Roma, Italy; iole.cordone@ifo.it

**Keywords:** functional high risk, early relapse, multiple myeloma

## Abstract

Background: Early relapse in multiple myeloma (MM) is a major predictor of poor prognosis, regardless of cytogenetic risk or treatment intensity. Methods: Here we analyzed 1026 MM patients treated across 12 Italian hematology centers. FHR was defined as progression-free survival (PFS) ≤18 months in transplant-eligible (TE) and ≤12 months in non-transplant-eligible (NTE) patients. Logistic regression and ROC analysis were used to identify significant predictors of FHR and build a risk score. Results: FHR status was identified in 175 patients (17%). These patients had significantly shorter PFS (7 vs. 57.5 months) and overall survival (19 months vs. not reached; *p* < 0.001). FHR status was associated with higher median LDH, lower Hb level, higher creatinine level and lower platelets count. Modified EASIX formula was built by these significant continuous variables, to be tested in a logistic analysis: [(LDH × creatinine)/(Hb × PLT) × 100]. A significantly higher rate of FHR was found with a score > 2.0 (89% vs. 11%, *p* < 0.001). Multivariate logistic analysis selected the above formula, ECOG PS ≥ 2 and ISS III as factors associated with FHR. Scoring these variables according to OR, three groups of patients were segregated with a rate of FHR patients of 7%, 29.5%, and 63.5%, respectively. Treatment with anti-CD38 monoclonal antibodies was associated with lower FHR frequency. Conclusions: This study proposes a simple, clinically applicable model to identify FHR MM patients early in their disease course. However, very in-depth biological tools, not available in clinical practice, are needed to identify singularly risk of becoming FHR.

## Simple Summary

This study examined 1026 multiple myeloma patients treated in 12 Italian centers to identify those at high risk of early relapse, known as functional high-risk (FHR). They had much shorter outcome measures compared to others. We found that higher LDH and creatinine levels, and lower hemoglobin and platelet counts, were linked to FHR. Using these values, we created a predictive formula similar to EASIX. Patients with a score above 2.0 were far more likely to be FHR. Additional risk factors included poor physical condition (ECOG ≥2) and advanced disease stage (ISS III). A scoring system grouped patients into low, intermediate, and high risk, with increasing FHR rates. Importantly, treatment with anti-CD38 monoclonal antibodies reduced the chance of early relapse. This model may help to identify high-risk patients early and tailor treatments to improve outcomes.

## 1. Introduction

The prognosis of multiple myeloma (MM) has significantly improved over the past decade thanks to the introduction of new drugs combined with monoclonal antibodies in multi-agent therapeutic regimens. However, MM is a highly heterogeneous disease from both a biological and prognostic standpoint, with survival ranging from a few months to over 10 years. Since the initial prognostic staging attempt by Durie & Salmon [1], several advances have been made, moving to the ISS stage [2], which, through β2-microglobulin and albumin levels, better represents the disease burden, then to the R-ISS stage [3], which combines ISS with LDH level, a marker of tumor replication, and cytogenetic abnormalities as del(17p); t(4;14) and t(14;16), expression of the biological aggressiveness of MM; and finally to the R2-ISS stage [4], incorporating 1q abnormalities as poor prognostic factors. More recently, baseline risk stratification has improved by the publication of the IMS/IMWG consensus recommendations on high-risk MM, establishing as high-risk a patient harboring ≥ 2 high-risk cytogenetic features, except for del(17p), biallelic 1(p32), and TP53 mutations that are sufficient alone [5]. Going beyond the mere cytogenetic definition, other factors are configured as relevant in risk stratification, as extramedullary disease (EMD) [6,7] and the presence of circulating plasma cells (CTC) at a cut-off not yet established [8,9,10]. However, patient-related variables such as age, performance status (PS), and frailty also play an important role in defining the prognosis of MM.

Although staging systems are valid, they describe the prognosis of groups of patients with similar characteristics and are not able to accurately predict the prognosis of an individual patient, especially when patients fall into intermediate stages of the scoring systems to which most patients belong. Approximately 10–20% of MM patients, not classified as high-risk by the above definitions, still have a progression-free survival (PFS) of less than 12–24 months despite receiving optimal first-line treatment. These patients are currently defined functional high-risk (FHR) and exhibit particularly poor survival (less than 3 years), often worse than that of patients traditionally defined as high-risk [11].

Molecular sequencing techniques may help identify these patients [12], but their application in clinical practice is currently limited to only a few centers. On the other hand, it would be extremely valuable to know in advance the probability of a patient developing FHR status during therapy or follow-up, as this could have important implications for patient counseling and therapeutic decision-making.

However, the method for identifying such patients should be quick and easy to implement in routine clinical practice, where cytogenetics and other advanced techniques are often not readily available. Furthermore, the method should be developed using unselected patients, unlike those in clinical trials, since trial populations do not accurately reflect the real-life setting.

The aim of this study is to assess whether it is possible to develop a risk model to predict FHR status using parameters that are readily available in clinical practice, in a large real-world population of patients treated with current therapies.

## 2. Materials and Methods

### 2.1. Study Design

MM patients treated from 2019 to 2024 in 12 hematological Italian centers were retrospectively analyzed. Clinical, laboratoristic, and cytogenetic features were collected according to the study plan in a dedicated database. The following parameters were considered: gender, age (linear and <65 vs. ≥65 years), the Eastern Cooperative Oncology Group (ECOG) performance status (<2 vs. ≥2), hemoglobin (linear and <10 vs. ≥10 g/dL), platelets count (linear and <150 vs. ≥ 150 × 10^9^/L), creatinine (linear and <1.2 vs. ≥1.2 mg/dL), albumin (linear and <3.5 vs. ≥3.5 g/dL), lactate dehydrogenase (LDH) (linear and normal or higher than the upper limit of the normal range), the international staging system (ISS), revised-ISS (R-ISS), and the presence versus absence of the high-risk cytogenetic alterations such as del(17p), t(4;14), t(14;16), amplification (1q), del(1p). FISH was performed in CD138-selected bone marrow plasma cells.

Patients with a PFS duration ≤ 18 months if transplant-eligible(TE) and ≤ 12 months if not-transplant-eligible (NTE), independently of traditional staging systems, were defined as FHR according to 33rd percentile of global median PFS of NTE (34 months) and TE (56 months).

Patients who died for reasons other than disease progression were excluded from the analysis.

The study was approved by the Institutional Review Board of each participating institution and was conducted in accordance with the Declaration of Helsinki.

### 2.2. Statistical Analysis

Continuous variables were presented by median and range, while those discrete by number and percentage. The former were compared by Mann–Whitney test, the latter by Chi-square test. PFS was defined as the time of study entrance until disease progression or death from any cause, while OS was defined as the time of study entrance until death from any cause.

According to the aim of this study, we firstly searched laboratory parameters significantly associated with FHR status by linear regression and then we combined them in a formula like Endothelial Activation and Stress Index (EASIX) [13] evaluated based on log2 transformed values. Optimal cut-off of this formula value was searched by ROC curve. Factors associated with FHR status were searched by logistic regression univariate and multivariate analysis. A *p* value of 0.1 was established as the significance level for removal from the model. Assigning an OR ratio-based score for each significant factor, we fitted a model to assess FHR cumulative incidence. The stability of regression model was evaluated through non-parametric bootstrapping (*n* = 1000 resamples), allowing estimation of bias-corrected confidence interval of the OR. Survival curve was performed by Kaplan–Meier methods and compared by log-rank test. Any *p* values less than 0.05 were considered statistically significant.

Statistical analysis were performed by SPSS statistics version 29 package.

## 3. Results

**Figure 1:**
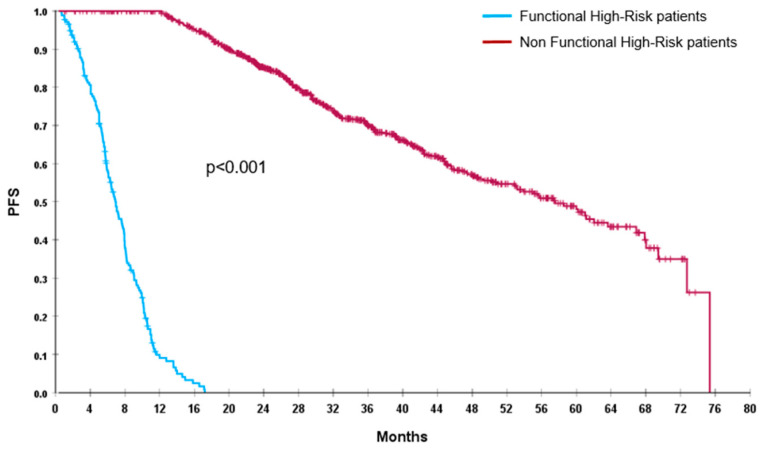
PFS of FHR (blue line) and non-FHR (red line) patients.

**Figure 2:**
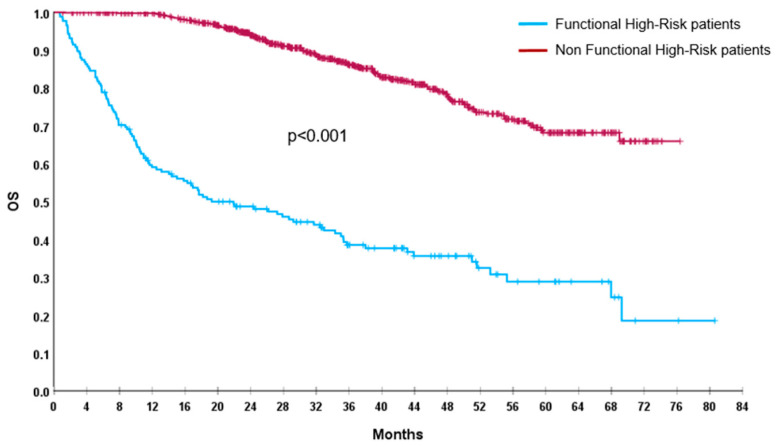
OS of FHR (blue line) and non-FHR (red line) patients.

Out of 1026 enrolled patients, by definition, we found 175 (17%) FHR patients, 72 (7%) were TE and 103 (10%) NTE. After a median follow-up of 41 months (95%CI: 38.5–42), median PFS of the global population was 45.5 months (95%CI: 40.5–50.5) and median OS was not reached (NR). FHR patients had a significantly shorter PFS (7 vs. 57.5 months; *p* < 0.001) and OS (19 months vs. NR; *p* < 0.001), compared with non-FHR population (Figure 1 and Figure 2).

The characteristics of the overall and the two populations (FHR and non-FHR) are pictured in Table 1.

Regarding therapy, 451 patients (44%) received anti-CD38-based frontline therapies, 525 (56%) received PI-based and/or IMID-based therapies (Table 2).

FHR status was associated with higher median LDH level (188 vs. 170; *p* = 0.013), higher median creatinine level (1.20 vs. 0.94; *p* = 0. 023), lower median Hb level (10.5 vs. 11.1; *p* = 0.004), and lower median platelets count (203 vs. 212; *p* = 0.034) as continuous variables. By these significantly continuous variables, the following formula, like EASIX, was built to test in a logistic analysis: Lg2[(LDH × creatinine)/(Hb × PLT) × 100]. A significantly higher rate of FHR was found with a score > 2.0 compared with lower one (89% vs. 11%: *p* < 0.001). Univariate logistic analysis selected the following variables as significantly affecting FHR status (Table 3): age > 70 years (OR: 1.2; *p* = 0.037), ECOG PS ≥ 2 (OR: 2.03; *p* < 0.0001), ISS III (OR: 1.46; *p* = 0.032), R-ISS III (OR: 1.1; *p* = 0.030), high-risk cytogenetics (OR: 1.52; *p* = 0.014), renal insufficiency (OR: 1.10; *p* = 0.038), and the above EASIX-modified formula (EmFormula) (OR: 1.81; *p* = 0.014). Multivariate stepwise logistic analysis retained ECOG PS ≥ 2 (OR: 2.22; *p* < 0.0001), ISS III (OR: 1.59; *p* = 0.007) and EmFormula > 2 (OR: 1.76; *p* = 0.001) as factors significantly associated with FHR status. The estimated bias by bootstrap resampling was pictured in Table 3.

Scoring these variables according to OR (2 points for ECOG PS ≥ 2, 1.5 points for both EmF > 2 and ISS III), three groups of patients were segregated: low-risk (LR: 0–1.5 points), intermediate-risk (IR: 2–3.5 points) and high-risk (HR: 5 points). The rate of FHR in these three risk categories was 7%, 29.5%, and 63.5%, respectively (*p* < 0.001) (Table 4). Our score is able to reclassify as FHR 9% of patients with ISS I, 20% with ISS II, and 30% of those with low-risk cytogenetics. However, the ROC AUC of the model was 65%.

Forcing frontline treatment as in the multivariate analysis, combinations containing anti-CD38 monoclonal antibodies results independently associated with lower FHR frequency (OR: 0.5; 95%CI: 0.38–0.73; *p* = 0.001). Therefore, we explored their effect on the risk groups of becoming FHR. Anti-CD38-containing therapies significantly reduce the rate of FHR both in the HR (30.5 vs. 65.5%: *p* < 0.0001) and in L-IR groups (40 vs. 60%; *p* = 0.042). The median PFS was 35.5 vs. 23 months in the HR group (*p* = 0.002) and it was 57.5 vs. 47 months in the L-IR group (*p* = 0.011) in patients treated or not with anti-CD38 monoclonal antibodies, respectively.

## 4. Discussion

The prognosis of patients with multiple myeloma (MM) has significantly improved over the past 10 years thanks to new therapies. However, high-risk patients, as defined by recent staging systems such as R-ISS [3] and R2-ISS [4] staging systems, continue to have a poor prognosis, as no therapy has yet been able to overcome this clinical-biological condition. Furthermore, a new category of patients with very unfavorable prognosis has recently emerged, the so-called FHR patients, who relapse early regardless of whether they are classified as high-risk by classical and modern prognostic criteria [11,12,14].

According to published studies, this group of patients accounts for between 10% and over 30% of cases, depending on the definition used, as the PFS used to classify FHR patients ranges from 12 to 24 months, with or without distinction between TE and NTE patients [15,16,17,18,19,20,21,22,23,24,25,26,27]. In our case, we chose to define FHR patients as those with a PFS below the 33rd percentile of the global median, distinguishing between TE and NTE patients. This choice is due to the evidence that such a threshold should not be considered fixed, as it must evolve in response to advances in available therapies. Using a percentile-based approach rather than an absolute value allows for dynamic adjustment that reflects improvements in treatment efficacy over time. However, our FHR patients were those with a PFS of <12 months (NTE: overall median PFS 34 months) and <18 months (TE: overall median PFS 56 months) overlapping with those reported in the literature [15,16,17,18,19,20,21,22,23,24,25,26,27]. Using this criterion, our study showed that FHR patients represented 17% of the entire cohort, a figure in line with other published studies [11,17,19,28,29]. These FHR patients had a PFS of only 7 months compared to 57 months for non-FHR patients, and an overall survival (OS) of just 19 months versus not reached in the non-FHR group.

Most studies conducted and published to date included patients from clinical trials or single-center cohorts [15,16,17,18,19,20,21,22,23,24,25,26,27,28,29,30]. However, it is well known that patients in clinical trials are highly selected, particularly in terms of performance status (PS), renal function, blood counts, and rapidly progressive disease that does not allow for the long screening process. Single-center cohorts, on the other hand, suffer from limited size and variability.

We conducted this analysis on a large, multicenter cohort of patients treated in real-life settings (only 8% in clinical trials) in recent years with modern therapies, including anti-CD38 monoclonal antibodies, which were not present in any previously published studies. The main limitation of our cohort is the presence of numerous missing data, especially regarding cytogenetics and, therefore, also R-ISS. However, in clinical practice, cytogenetic analysis is often unavailable, delayed, or simply not performed, particularly in NTE patients, since treatment is not tailored based on cytogenetic risk [31]. Several studies have attempted to identify FHR patients using even more sophisticated genomic methods [12], which, however, are not available in most hematology centers. It would therefore be desirable to identify FHR patients in advance using easily accessible clinical and laboratory parameters.

Our study showed that LDH, creatinine levels, hemoglobin, and platelet count, when combined in a specific formula, were significantly associated with the likelihood of becoming FHR during follow-up after the start of therapy. Regarding the disease itself, a high ISS was predictive of a significantly increased risk of becoming FHR. Elevated serum LDH levels have long been recognized as markers of higher tumor burden, higher proliferation rate, and, ultimately, greater disease aggressiveness, leading to a poor prognosis [32,33]. From a biological standpoint, LDH has been shown to promote tumor growth, angiogenesis, as well as tumor cell invasion and migration [34]. Moreover, it appears to contribute to the immunosuppressive nature of the tumor microenvironment by impairing cytotoxic T lymphocyte and natural killer (NK) cell function, while promoting the presence of tumor-associated macrophages, myeloid-derived suppressor cells, and regulatory T cells expressing PD-1 [35,36,37]. Renal impairment has long been recognized as a negative prognostic factor, as confirmed by a meta-analysis by Mohyuddin et al. [38] in which patients with renal failure showed higher relative risk of progression or mortality if compared with those without renal failure. It is important to emphasize that the negative prognostic impact of renal insufficiency at diagnosis persists even in patients who subsequently experience recovery of renal function [39]. Platelet count and hemoglobin levels represent indirect indicators of tumor burden and reductions in platelets count have been associated with early morbidity and mortality [40,41,42]. From a pathogenetic perspective, hematopoietic stem and progenitor cells (HSPCs), particularly megakaryocytice-erythrocyte progenitors, are significantly reduced in the bone marrow of patients with MM. This reduction is attributable not only to infiltration of malignant plasmacells but also to an underlying functional impairment [43]. Moreover, the number of bone marrow HSPCs appears to be inversely correlated with the number of CD138+ cells, and the bone marrow microenvironment may suppress differentiation, thereby contributing to the development of anemia [44]. It is quite intuitive, in any case, that a patient with multiple genetic/genomic high-risk alterations, extramedullary disease, and circulating plasma cells would be at risk of becoming FHR. Nonetheless, it is of interest that our scoring system is capable of identifying as FHR approximately 30% of patients with ISS < III, as well as a comparable proportion of those with non-high-risk cytogenetics.

As shown in other studies, including the Spanish study and others [11,29], our study also found that poor ECOG performance status is independently associated with a higher likelihood of being FHR. This is not surprising, as it reflects the patient’s functional status in relation to disease burden, especially bone and organ involvement. Moreover, it influences the intensity of therapy delivered.

By combining these factors, we were able to identify three risk groups with significantly different rates of FHR MM. Specifically, patients with no risk factors had a very low probability of becoming FHR (<10%), while nearly two-thirds of those with all risk factors were likely to become FHR. The intermediate-risk group, as usual, is where the most uncertainty lies; nonetheless, fewer than one-third of these patients are at risk of becoming FHR. However, the model’s performance does not exceed 65%, as shown by the ROC curve, although bootstrapping bias was very low, indicating minimal deviation from the original estimate and suggesting that the statistic is nearly unbiased.

Zaccaria et al. [28], using data from 2190 newly diagnosed MM patients enrolled in phase II/III clinical trials, developed the Simplified Early Relapse Score (S-ERMM) based on six parameters weighted according to a score: 5 points for high LDH level or the presence of t(4;14); 3 points for the presence of del17p, abnormal albumin and bone marrow plasma cells more than 60%; 2 points for the presence of λ FLC. Three groups of patients were identified (low-risk with a total score ≤5; intermediate-risk with a score between 6 and 10; high-risk with a score ≥11) with significantly different risk of early relapse (ER18) (intermediate- vs. low-risk group OR = 2.39, *p* < 0.001; high- vs. low-risk group OR = 5.59, *p* < 0.001). The AUC of this model was 66%, but in a recent external validation using data of 476 patients from the CoMMpass study, AUC was found to be 59%, lower than that previously reported [45]. In the Spanish study [29], the model to predict early relapse (ER18) was built with ECOG PS, ISS, LDH, calcium level, bone marrow plasma cells, and a modified high-risk IMS classification [5], not including TP53 mutation and biallelic del (1p32). In this case as well, by assigning a score to these features, three patient groups were identified with an ER18 risk of 2%, 24.5%, and 59% in the low-, intermediate-, and high-risk groups, respectively. The performance of the model in terms of AUC was 70%, but it should be validated in an independent cohort of patients. Both scores were developed using clinical data from patients enrolled in clinical trials who did not receive the most modern combinations, particularly those containing anti-CD38 monoclonal antibodies. Furthermore, both models include cytogenetic data which, as previously mentioned, are not always available since they are not routinely performed in all patients [31].

It is evident that a portion of patients who become FHR cannot be identified in advance, even with the most sophisticated methods, as this phenomenon can be dynamic, potentially induced by the selective pressure of therapy on the disease or microenvironment, leading to the emergence of particularly aggressive and resistant clones. This dynamic nature has been addressed in some studies by including response to therapy after a certain period of time [17,19,26,30], but this is heavily affected by immortal time bias being time-of-response-unpredictable. On the contrary, frontline therapy is a milestone of outcomes and easily recognized. In this regard, the most original finding of this study is the impact of initial therapy with combinations containing the anti-CD38 monoclonal antibody on the frequency of FHR in different risk groups, a finding not reported elsewhere in the literature [46]. High-risk patients treated with these therapies had more than a 50% reduction in FHR frequency compared to those treated without anti-CD38 agents, while those in the low-intermediate risk group had a 20% reduction. This finding underscores the importance of a therapy capable of achieving a rapid and deep response in significantly reducing the emergence of resistant clones.

While the availability of predictive models is essential, it is equally important to have appropriate therapeutic strategies for FHR patients. Several prospective studies have been conducted specifically for high-risk patients defined by cytogenetics, as phase III GMMG CONCEPT [47], phase II IFM 2018-04 [48], phase II OPTIMUM (MUKnine) trials [49], and several ongoing trials are evaluating different MRD-adapted therapeutic strategies, considering that MRD negativity is becoming one of the best predictors of PFS and OS in MM [50]. MRD status has been used to discontinue consolidation therapy, as in the phase II MASTER trial [51]; to guide decisions regarding post-induction autologous stem cell transplantation, as in the phase III MIDAS study [52]; and to de-escalate maintenance therapy, as demonstrated in the PERSEUS trial [53]. Additional ongoing studies, such as the DRAMMATIC and RADAR trials, are investigating the feasibility of discontinuing maintenance therapy in patients who achieve sustained MRD negativity. Prospective studies focusing on FHR disease remain extremely limited, but two studies are particularly noteworthy for their use of chimeric antigen receptor T-cell (CAR-T) therapy. A 2-year progression-free survival (PFS) rate of 73% was achieved with cilta-cel in patients experiencing early relapse (≤12 months) after autologous stem cell transplantation (ASCT) in Cohort B of the CARTITUDE-2 study [54]. Additionally, a median PFS of 11.4 months was reported in patients who relapsed within 18 months of initial therapy in Cohort 2a of the KarMMa-2 trial [55].

Our study presents several important limitations, including its retrospective design, the incompleteness of certain data, particularly cytogenetic information and other currently relevant parameters such as updated risk scores, circulating plasma cells, and minimal residual disease (MRD), as well as treatment heterogeneity and recently approved quadruplets combination therapies. Additionally, external and/or prospective validation is warranted.

## 5. Conclusions

Outside of patients with extramedullary disease, circulating plasma cells, and complex cytogenetic/genomic alterations, where early relapse can be largely anticipated, our study demonstrates that simple clinical and promptly available laboratory parameters, appropriately combined, can predict in advance a substantial proportion of patients likely to become FHR, although our score still needs external validation. We could therefore suggest applying our score in situations where more sophisticated biological parameters are not readily available. Modern therapies, particularly combinations containing anti-CD38 monoclonal antibodies, appear to greatly reduce the frequency of early relapses. Therefore, patients at high risk of becoming FHR should be treated with these combinations. It is foreseeable that in the near future, the proportion of FHR patients, as currently defined, will significantly decrease, though it is likely that they will be redefined using longer PFS cut-offs, as PFS is expected to progressively extend with the introduction of novel therapies.

## Figures and Tables

**Table 1:** Characteristics of patients.

Characteristics	Total = 1026 N (%)	FHR = 175 N (%)	Non-FHR = 851 N (%)	*p*
Age, median (range), yrs	69 (27–93)	72 (42–93)	69 (27–90)	0.126
Age				
≤70 years	566 (55)	84 (48)	482 (56.5)	0.037
>70 years	460 (45)	91 (52)	369 (43.5)	
Sex				
Male	569 (55.5)	94 (54)	475 (56)	0.738
Female	455 (45.5)	79 (46)	376 (44)	
ECOG PS				
<2	703 (73)	65 (39.5)	638 (80)	<0.0001
≥2	257 (27)	99 (60.5)	158 (20)	
Missing	66			
LDH, median (U/L)	171	188	170	0.013
LDH (U/L)				
Normal	724 (76)	45 (28)	679 (86.5)	<0.0001
>Normal	224 (24)	116 (72)	108 (13.5)	
Missing	78			
PLT, median (× 10^9^/L)	211	203	212	0.034
PLT(×10^9^/L)				
≥150	751 (73.5)	46 (26.5)	705 (83)	0.003
<150	270 (26.5)	128 (73.5)	142 (17)	
Hb, median (g/dL)	11	10.5	11.1	0.004
Hb (g/dL)				
≥10	660 (65)	71 (41)	589 (70)	0.007
<10	360 (35)	103 (59)	257 (30)	
Creatinin, median (mg/dL)	1.0	1.2	0.94	0.023
Creatinin (mg/dL)				
<2	792 (78)	121 (70)	671 (80)	0.008
≥2	221 (22)	51 (30)	170 (20)	
Albumin, median (g/dL)	3.7	3.6	3.8	0.038
Extramedullary disease				
Yes	47 (5)	7 (4)	40 (5)	0.721
No	916 (89)	135 (77)	658 (77)	
Missing	63 (6)	33 (19)	153 (18)	
FISH analysis				
SR	431 (67)	38 (38)	393 (72)	0.008
HR	212 (33)	62 (62)	150 (28)	
Missing	383			
ISS				
I	349 (35)	30 (18)	319 (39)	<0.0001
II	337 (34)	65 (39)	272 (33)	
III	305 (31)	73 (43)	232 (28)	
Missing	35			
R-ISS				
I	197 (32)	13 (13.5)	184 (35.5)	<0.0001
II	315 (51.5)	52 (43)	263 (51)	
III	102 (16.5)	33 (34)	69 (13.5)	
Missing	412			

**Table 2:** Initial therapies of 1026 patients.

Therapies	FHR = 175 N (%)	Non-FHR = 851 N (%)
Anti-CD38 plus bortezomib-based	28 (16)	228 (27)
Bortezomib plus thalidomide-based	41 (24)	212 (25)
Anti-CD38 plus lenalidomide-based	23 (13)	153 (18)
Bortezomib-based	46 (26)	147 (17)
Lenalidomide-based	28 (16)	76 (9)
Anti-CD38 plus lenalidomide and bortezomib-based	2 (1)	16 (2)
Bortezomib plus lenalidomide-based	0	12 (1.5)
Carfilzomib-based	0	3 (0.3)
Others	7 (4)	4 (0.4)

**Table 3:** Univariate and multivariate analyses for FHR status.

	Univariate		Multivariate		Bootstrapping Bias
Characteristics	OR	*p*	OR (95% CI)	*p*	
Age > 70 years	1.20	0.037			
ECOG PS ≥ 2	2.03	0.001	2.22 (1.59–3.10)	<0.0001	−0.005
ISS III	1.46	0.032	1.59 (1.22–2.22)	0.007	0.013
R-ISS III	1.10	0.030			
FISH HR	1.52	0.014			
Renal failure	1.10	0.038			
EmFormula > 2	1.81	0.014	1.76 (1.24–3.04)	0.001	0.021

**Table 4:** Rate of FHR MM in the risk groups based on ECOG PS, ISS, and EmFormula.

Risk	Points	FHR Rate (%)	*p*
Low	0–1.5	7	<0.001
Intermediate	2–3.5	29.5	
High	5	63.5	

## Data Availability

The data presented in this study are available on request from the corresponding author. The data are not publicly available due to privacy restrictions.
